# Supplementary figures and images for: The influence of oviposition status on measures of transmission potential in malaria-infected mosquitoes depends on sugar availability
Source: Parasit Vectors. 2024 May 23;17:236. doi: 10.1186/s13071-024-06317-2 (PMC11118549; doi:10.1186/s13071-024-06317-2)

**A**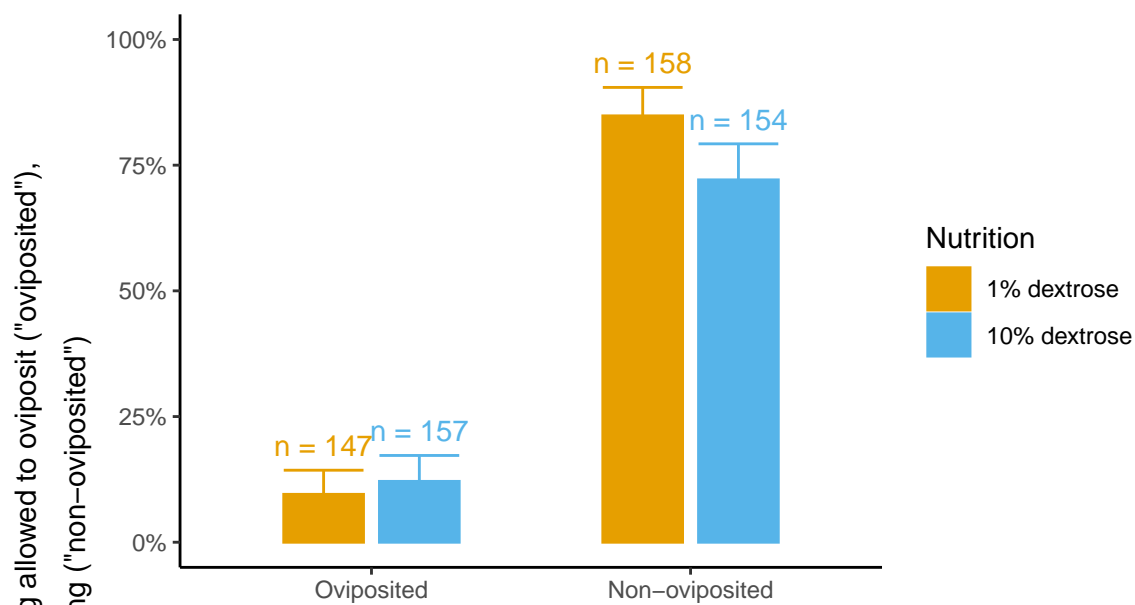**B**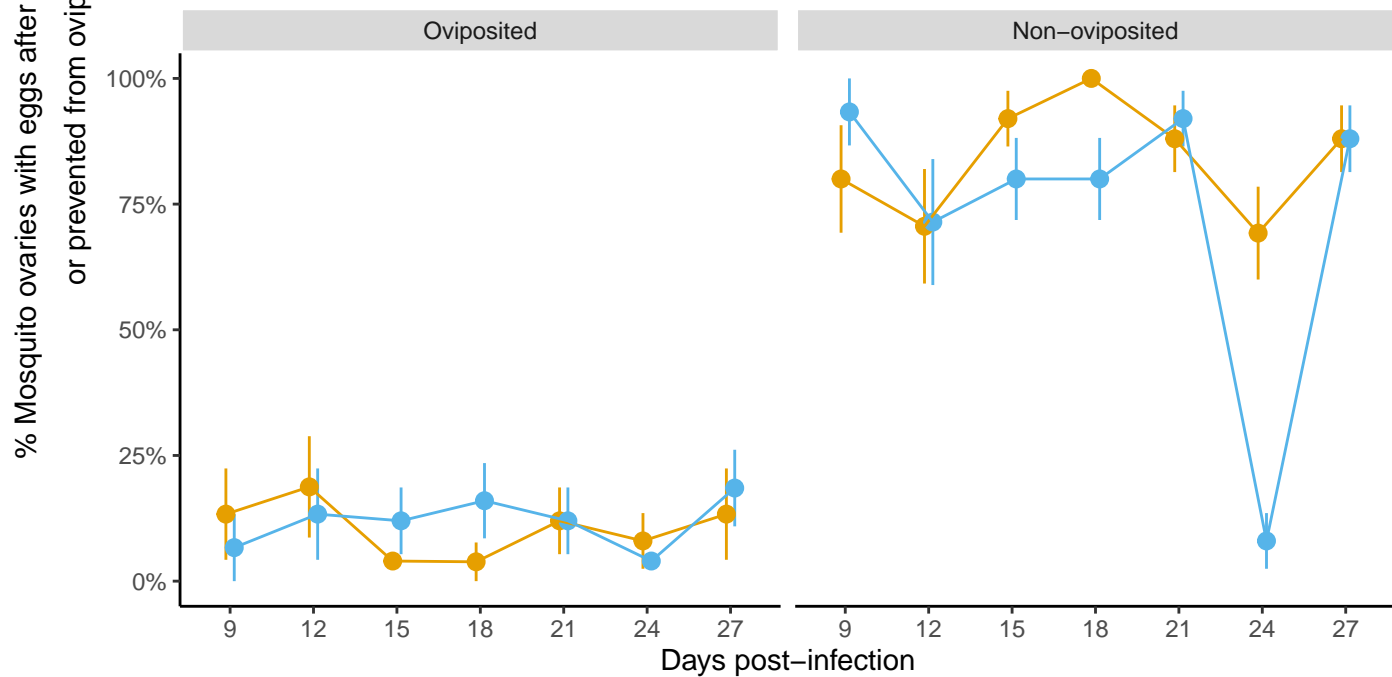

Supplement: Supplementary file 1 — Additional file 1: Fig. S1. (A) The proportion of mosquitoes that successfully oviposited (proportion with no eggs in ovaries) after being provided an oviposition site (left pane, ‘Oviposited’) is unaffected by time or sugar concentrations (‘Nutrient levels*dpi’, χ2 = 0.22, df = 1, P = 0.638, Additional file 3: Table S1); in non-oviposited mosquitoes, the proportion of mosquitoes still retaining eggs appears to be influenced by dextrose levels (‘Nutrient levels’, χ2 = 6.86, df = 1, P = 0.009, Additional file 3: Table S1), although this effect is primarily due to the data point at 24 dpi in the non-oviposited mosquito group fed 10% dextrose: excluding this data point eliminated this apparent effect of nutrient treatment (‘Nutrient levels’, χ2 = 0, df = 1, P = 0.9686). (B) Mean rates of gravidity recorded at the time points in A) with sample sizes of each indicated above the data; note that the sum of these values (147 + 157 + 158 + 154) total 616, which is the number of mosquitoes assessed for gravid status at the time of dissection, with 416 sampled while estimating rates of sporozoite prevalence (Fig. 2) and 200 while quantifying sporozoite densities (Fig. 3). For statistical analysis of the data, refer to Table S1. [file 13071_2024_6317_MOESM1_ESM.pdf]
