# Supplementary material for: The influence of oviposition status on measures of transmission potential in malaria-infected mosquitoes depends on sugar availability
Source: Parasit Vectors. 2024 May 23;17:236. doi: 10.1186/s13071-024-06317-2 (PMC11118549; doi:10.1186/s13071-024-06317-2)
Supplement: Supplementary file 3 — Additional file 3: Figure S2. Excluding individuals that did not oviposit when offered an oviposition site or oviposited despite not being offered an oviposition sites (i.e. non-responders, dot-dash lines) does not influence the overall trends in sporozoite prevalence (A) or densities (B). To ensure compatibility with the vector survival data where it was not possible to discriminate oviposition status of dead mosquitoes, this similarity and the generally high correlation between the means (Pearson’s correlation ≥ 0.99) meant all statistical analyses were performed with datasets that consider all individuals irrespective of whether they responded to the oviposition sites as expected (true and non-responders together in all groups). [file 13071_2024_6317_MOESM3_ESM.pdf]

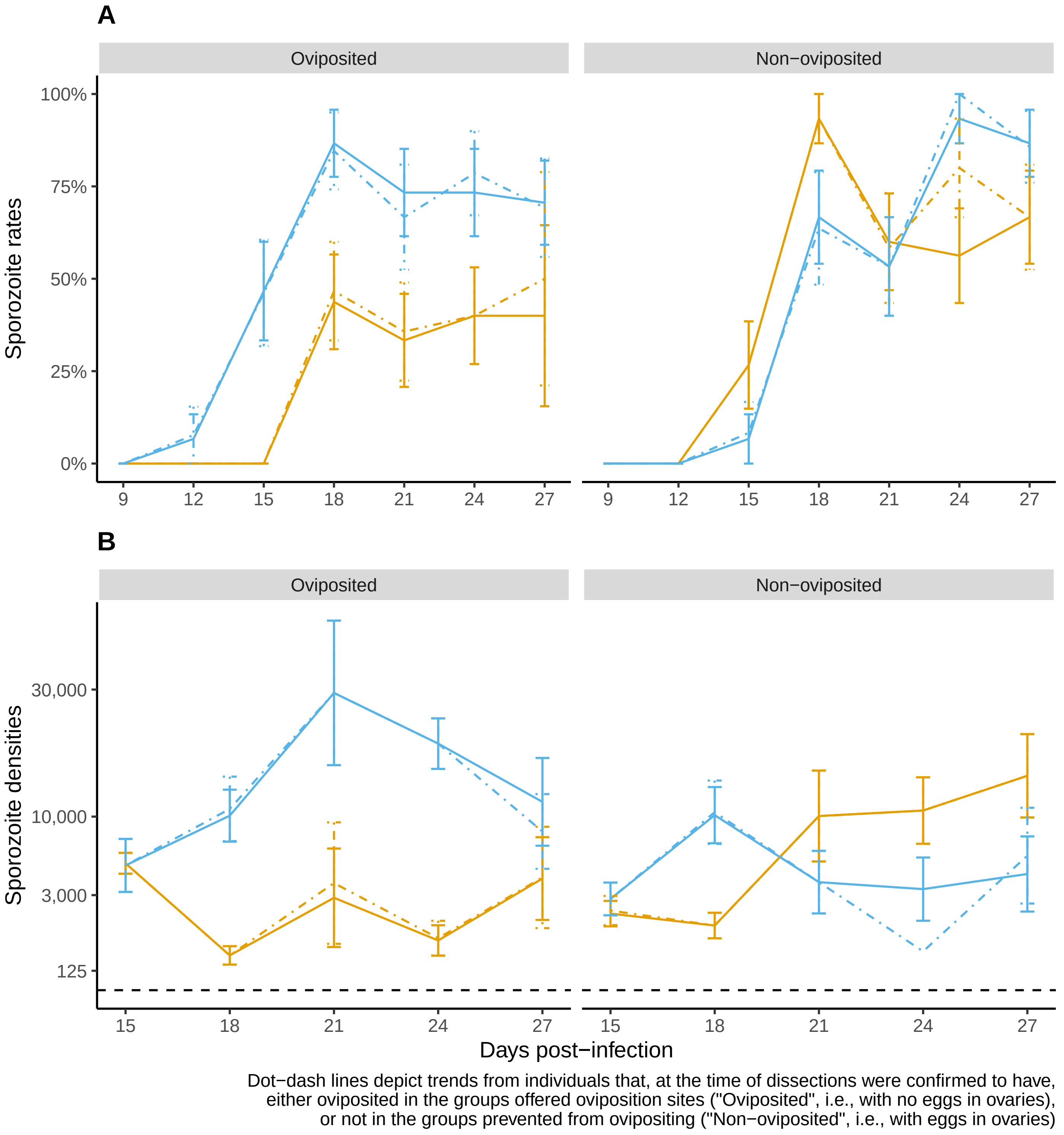

A
Oviposited
Non−oviposited
100%
75%
50%
25%
0%
Sporozoite rates
9
12
15
18
21
24
27
B
Oviposited
Non−oviposited
30,000
10,000
3,000
125
Sporozoite densities
15
18
21
24
27
Days post−infection
Dot−dash lines depict trends from individuals that, at the time of dissections were confirmed to have, either oviposited in the groups offered oviposition sites ("Oviposited", i.e., with no eggs in ovaries), or not in the groups prevented from ovipositing ("Non−oviposited", i.e., with eggs in ovaries)
