# Supplementary material for: The influence of oviposition status on measures of transmission potential in malaria-infected mosquitoes depends on sugar availability
Source: Parasit Vectors. 2024 May 23;17:236. doi: 10.1186/s13071-024-06317-2 (PMC11118549; doi:10.1186/s13071-024-06317-2)
Supplement: Supplementary file 6 — Additional file 6: Table S4. Statistical model of survival rates (Fig. 3). Survival was measured daily in all four groups until 27 dpi and data modeled using Cox proportional hazards. Mosquitoes that were destructively sampled for parasite quantification were considered censored in the analysis. [file 13071_2024_6317_MOESM6_ESM.docx]

| **Table S4** | | | | |
| --- | --- | --- | --- | --- |
|  |  | *y* = daily mortality risk | | |
| Row | Terms (*x*) | *χ^2^* | *df* | *P* |
| 1 | Oviposition status ^a^ | 15.37 | 1 | <0.001* |
| 2 | Nutrient levels ^b^ | 4.94 | 1 | 0.026* |
| 3 | Oviposition status * nutrient levels | 2.36 | 1 | 0.124 |
| ^a^ Oviposited or Non-oviposited mosquitoes; ^b^ 1 or 10% dextrose. | | | | |
| Abbreviations:  *χ^2^*= Chi-squared test (type 2); *df* = degrees of freedom; *P* = p-value. | | | | |
